# Serologic and Histologic Predictors of Long-Term Renal Outcome in Biopsy-Confirmed IgA Nephropathy (Haas Classification): An Observational Study

**DOI:** 10.3390/jcm8060848

**Published:** 2019-06-14

**Authors:** Shang-Feng Tsai, Ming-Ju Wu, Mei-Chin Wen, Cheng-Hsu Chen

**Affiliations:** 1 Division of Nephrology, Department of Internal Medicine, Taichung Veterans General Hospital, Taichung 407, Taiwan; s881056@gmail.com (S.-F.T.); ymdoctor@hotmail.com (M.-J.W.); 2 Department of Life Science, Tunghai University, Taichung 407, Taiwan; 3 School of Medicine, National Yang-Ming University, Taipei 112, Taiwan; 4 Department of Pathology, Taichung Veterans General Hospital, Taichung 407, Taiwan; mewen@vghtc.gov.tw

**Keywords:** IgA nephropathy, patient outcome, renal outcome

## Abstract

*Background and objective*: The Haas classification of IgA nephropathy should be validated for Asian populations. More detailed and newer predictions regarding renal outcome of IgA nephropathy remains mandatory. *Materials*: We conducted a retrospective cohort study between January 2003 and December 2013. Clinical, Pathological, and laboratory data were all collected via available medical records. A Mann–Whitney U test was used for continuous variables and the Chi-square test was implemented for categorical variables. A Kaplan–Meier curve was put in place in order to determine patient survival and renal survival. The Youden index and Cox proportional hazard regression were used to investigate the possible factors for renal survival and predictive power. *Results*: All 272 renal biopsy-confirmed IgAN patients were enrolled for further studies. The univariate analysis showed that risk factors for poor renal outcome included stage 4–5 of Haas classification (HR = 3.67, *p* < 0.001), a poor baseline renal function (HR = 1.02 and *p* < 0.001 for higher BUN; HR = 1.14 and *p* < 0.001 for higher serum creatinine; HR = 0.95, *p* < 0.001 for higher eGFR), IgG ≤ 907 (HR = 2.29, *p* = 0.003), C3 ≤ 79.7 (HR = 2.76, *p* = 0.002), a higher C4 (HR = 1.02, *p* = 0.026), neutrophil-to-lymphocyte ratio > 2.75 (HR = 2.92, *p* < 0.001), and a platelet-to-lymphocyte ratio ≥ 16.06 (HR = 2.02, *p* = 0.012). A routine-checked markers, such as neutrophil-to-lymphocyte ratio and platelet-to-lymphocyte ratio, in order to predict the renal outcome, is recommended. *Conclusions*: This is the first study to demonstrate that Haas classification is also useful for establishing predictive values in Asian groups. A lower serum IgG (≤907 mg/dL) and serum C3 (≤79.7 mg/dL) were both risk factors for poor renal outcome. Additionally, this is the first study to reveal that serum C4 levels, an NLR > 2.75 and a PLR > 16.06, S could suggest poor renal outcome.

## 1. Introduction

Immunoglobulin A Nephropathy (IgAN) is the most common type of glomerulonephritis throughout the world [1], with a wide range of histologic patterns and complex clinical manifestations. Its outcome also involves a wide spectrum ranging from asymptomatic hematuria to end-stage renal disease (ESRD) [2]. Therefore, timely identification and treatment for this high-risk group is mandatory. Currently, certain clinical situations and histopathological features, including the degrees of glomerular and interstitial fibrosis, tubular atrophy, and glomerular hypercellularity, are considered independent risk factors for the progression of IgAN to ERSD. However, approximately 50% of these patients progress to ESRD even though they had undergone aggressive treatment for 20–25 years [3]. Undoubtedly, there still remain some undetected risk factors involving the progression of IgAN which need to be discovered. Thus, the aim of this study is to attempt to reveal any other undetected risk factors for ESRD of IgAN. 

There have been some established histopathological classifications for renal outcome prediction [4,5,6]. The Lee classification was published; however, mesangial sclerosis and segmental glomerulosclerosis were not clearly distinguished [4]. Subsequently, the Haas classification was used for the prediction of renal outcome but it did not specify either mesangial or endocapillary hypercellularity [5]. More recently, the Oxford classification has been used worldwide but it is unknown whether or not it can predict future adverse renal outcomes better than previous classifications [6,7]. Much controversy has been created as to whether histopathological features are superior to clinical factors in the risk stratification of IgAN [8]. There have been 22 validation studies of the Oxford classification [9]. Recently, Park el al. [7] suggested that the Haas and the Oxford classifications are comparable in predicting the progression of IgAN. However, much fewer studies have been performed in order to validate the previous Haas classification. In this study, we attempted to re-evaluate the predictive power of the Haas classification. 

The outcome of IgAN is heterogeneous, and timely identification of patients who are at a high risk of disease progression remains mandatory in clinical practice. We verified all available clinical and histological risk factors in this cohort of this studyin order to uncover any new risk factors. Additionally, models which combine clinical and pathological risks together in the Asian population are both lacking and without validation [10]. We also analyzed our data of this study in our institute as follows to create a new model to predict renal outcome.

## 2. Materials and Methods

### 2.1. Study Population

We conducted a retrospective cohort study between January 2003 and December 2013. Participants of an age > 20 years old who had undergone their first renal biopsy with the diagnosis of IgAN were enrolled in our medical center in Taiwan. Graft renal biopsies were excluded. This medical center possesses the largest patient population for those who have undergone renal biopsies (more than 8000 within the last 30 years). This study was approved by Ethics Committee of Taichung Veterans General Hospital, IRB number:CE15125B. All methods were carried out in accordance with relevant guidelines and regulations and informed consent was obtained from all subjects. 

### 2.2. Data Collection and Outcome Assessment

All data was obtained from this cohort via the reviewing of medical records. Baseline data was collected at the time of each patient’s renal biopsy, including gender, age, body height (cm), body weight (kg), and systolic or diastolic blood pressure (SBP and DBP). Data from a blood sample were also collected for blood urea nitrogen (BUN) (mg/dL), serum creatinine (mg/dL), estimated glomerular filtration rate (eGFR from MDRD equation [11]) (mL/min/1.73 m^2^), white blood cell (WBC) (/cumm), red blood cell (RBC) (/cumm), hemoglobin (g/dL), neutral and lymphocyte ratio (%), platelet count (/cumm), uric acid (mg/dL), sodium (meq/L), potassium (meq/L), calcium (mg/dL), phosphate (mg/dL), magnesium (mg/dL), albumin (g/dL), total protein (g/dL), glutamate oxaloacetate transaminase (GOT) (U/L), glutamate–pyruvate transaminase (GPT) (U/L), total cholesterol (mg/dL), triglyceride (mg/dL), low-density lipoprotein (LDL) (mg/dL), high-density lipoprotein (HDL) (mg/dL), fasting and postprandial blood sugar (mg/dL), glycated hemoglobin (%). Neutrophil-to-lymphocyte ratio (NLR) and platelet-to-platelet ratio (PLR) were calculated as the ratio of the neutrophil or the lymphocyte count to lymphocyte count. Chronic infection or inflammatory markers were included as follows; hepatitis B status, hepatitis C status, anti-nuclear Ab (ANA), anti-double stranded DNA (anti-dsDNA), anti-neutrophil cytoplasmic antibodies (ANCAs), proteinase 3 (PR3) and myeloperoxidase (MPO). Urine samples were tested for spot urine protein (mg/dL), spot urine creatinine (mg/dL), spot urine albumin (mg/dL), and 24 h proteinuria (g/day).

All pathological samples were analyzed by the same pathologist, while all enrolled participants had their diagnosis of IgAN based upon the Haas classification criteria [5]. The study endpoints were patient death and renal death (ESRD), those who needed the initiation of dialysis or those receiving transplantation according to local guidelines.

### 2.3. Statistical Methods

Data was expressed as the mean ± SD in continuous variables and as numbers (percentages) in categorical data. A Mann–Whitney U test was used for continuous variables and the Chi-square test was used for categorical variables. A Kaplan–Meier curve was implemented for measuring both patient survival and renal survival.

The Youden index was used for the capture of the results taken from a dichotomous diagnostic test for both renal and patients outcomes. The area under the curve of the receiver operating characteristic curve (AUC of ROC) means the predictive value. If an AUC value higher than 0.70, the prediction is perfection. If the AUC values between 0.7 and 0.5, the prediction would be considered as acceptable.

A Cox proportional hazard regression was used to analyze the possible factors for renal survival (both the univariable and multivariable Cox models). We first did univariate Cox regression to discover any possible factors. The results were 19 factors in the univariate Cox regression model. Initially, the results were 16 factors in the univariate Cox regression model with significance: age, cresentic GN, BUN, serum creatinine, eGFR, uric acid, albumin, total protein, urine PCR, IgG, C3, C4, NLR, PLR, 24 h proteinuria, and Haas classification. We then deleted apparent multi-collinearity. We chose eGFR rather than BUN and serum creatinine. We chose blood albumin rather than PCR and 24 h proteinuria. We also deleted the variable of age due to the minor increased hazard ratio only (1.03). Haas classification and crescentic GN were also deleted because of the already-known risk factors in many previous studies. Finally, we had nine factors for multivariate Cox regression analysis: eGFR, uric acid, albumin, total protein, IgG, C3, C4, NLR, and PLR. 

A value of *p* < 0.05 was considered statistically significant. All statistical procedures were performed using the SPSS statistical software package, version 17.0 (Chicago, IL, USA). 

## 3. Results

### 3.1. Pathology Regarded as an Important Influencing Factor According to the Haas Classification

Initially, 388 patients were enrolled in this study. We then excluded 53 renal biopsies for graft kidney biopsy, while also excluding 63 renal biopsies whose renal biopsy was their second or third biopsy. Ultimately, the 272 remaining renal biopsy-confirmed IgANs were enrolled for further studies. The whole duration of follow-up was 11 years (January 2003 to December 2013). The mean duration of follow-up was 7.2 ± 3.1 years. Based on the Haas classification system [4], we grouped all the participants into either the excellent-prognosis subgroup (subclass 1–2), or the intermittent to poor-prognosis subgroup (subclass 3–5) as shown in Table 1. As for the baseline clinicopathological and laboratory data, the participants in the moderate-to-advanced subclass of IgAN exhibited more crescent formation (*p* = 0.006), a poorer renal function (higher BUN (*p* < 0.0001)), more serum creatinine (*p* = 0.001) and lower eGFR (*p* = 0.006)), anemia (*p* = 0.008), hyperuricemia (*p* = 0.016), hypocalcemia (*p* = 0.001), and hypoalbuminemia (*p* < 0.001), along with lower blood total protein (*p* = 0.001), hyperphosphatremia (*p* = 0.037), and LDL (*p* = 0.005). Additionally, more proteinuria (*p* < 0.0001 of daily urine protein and *p* = 0.021 of urine PCR) or albuminuria (*p* = 0.022 of urine albumin and *p* = 0.0009 of urine ACR) was presented.

### 3.2. The Patients’ Outcome Analysis

**Figure 1:**
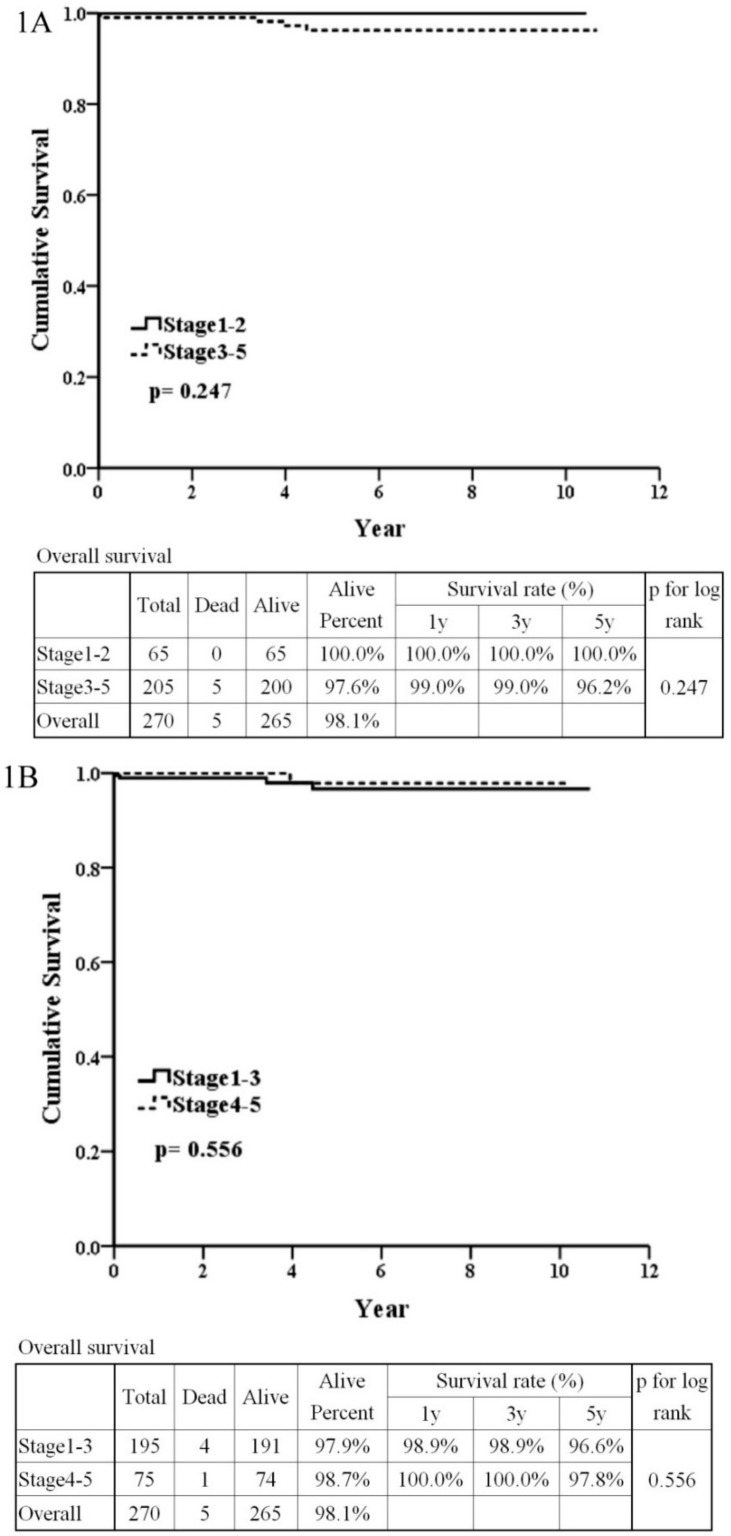

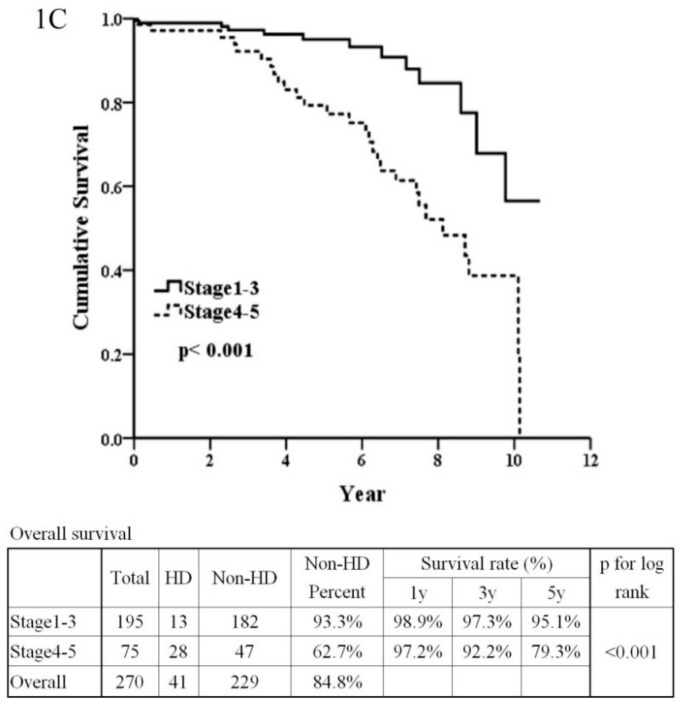
Patient and renal survival of IgA nephropathy according to Haas. (**1A**) Patient survival between patients with stage 1–2 and stage 3–5 IgA nephropathy. (**1B**) Patient survival between patients with stage 1–3 and stage 4–5 IgA nephropathy. (**1C**) Renal survival between patients with stage 1–3 and stage 4–5 IgA nephropathy.

**Figure 2:**
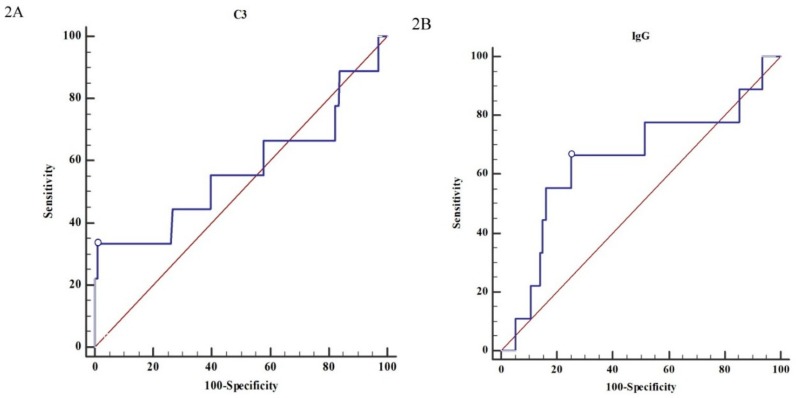

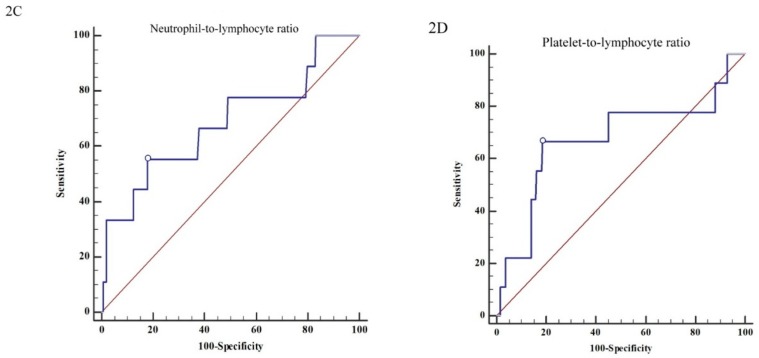
Predicting patients’ survival of IgA nephropathy with C3 level, IgG level, ratio of neutrophil/lymphocyte, and ratio of platelet/lymphocyte. (**2A**) C3 predicted patients’ survival with poor power (AUC = 0.568, sensitivity = 33.33%, specificity = 99.02%). (**2B**) IgG predicted patients’ survival with fair power (AUC = 0.648, sensitivity = 66.67%, specificity = 74.82%). (**2C**) Neutrophil-to-lymphocyte ratio predicted patients’ survival with fair power (AUC = 0.684, sensitivity = 55.56%, specificity = 82.08%). (**2D**) Platelet-to-lymphocyte ratio predicted patients’ survival with good power (AUC = 0.673, sensitivity = 66.67%, specificity = 81.54%).

The patients’ survival rates according to the Haas classifications were as follows: 100% for stage 1–2 and 96.2% for stage 3–5 (*p* = 0.247) (Figure 1A), or 96.6% for stage 1–3 and 97.8% for stage 4–5 (*p* = 0.556) (Figure 1B). There was no statistical significance of patients’ survivals between the Haas stages in Figure 1A,B. The predictive power of C3 (Figure 2A), IgG (Figure 2B), NLR (Figure 2C), and PLR (Figure 2D) was poor (AUC = 0.568, 0.648, 0.684, and 0.673, respectively) (Figure 2). 

### 3.3. The Renal Outcome Analysis

**Figure 3:**
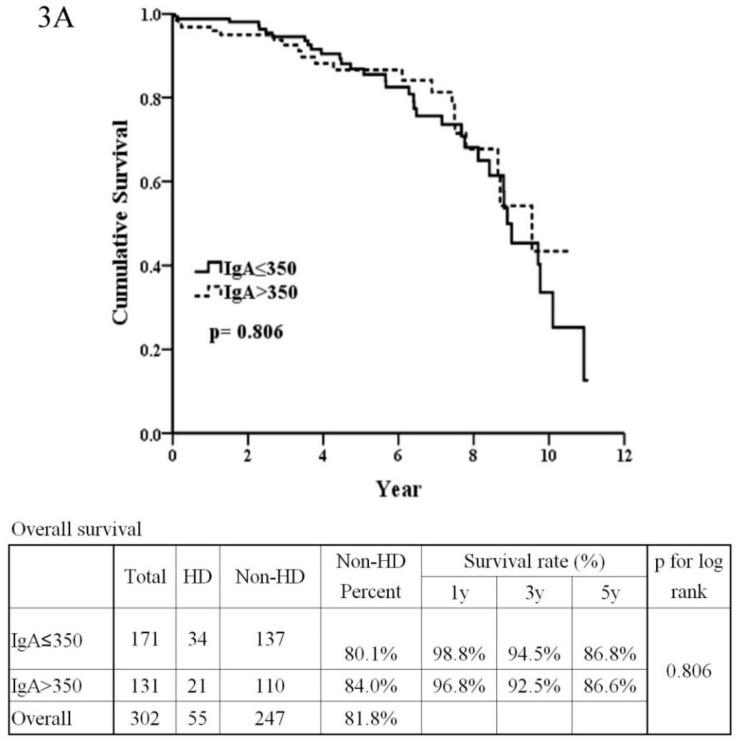

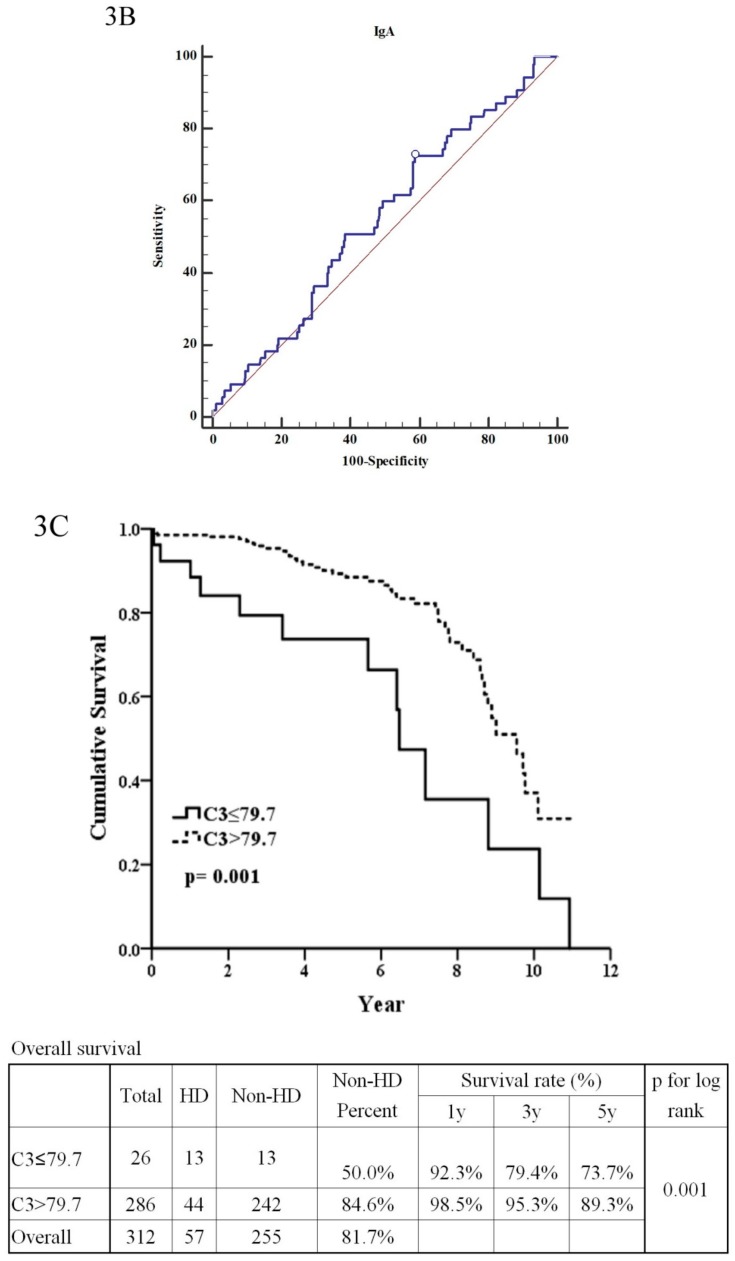

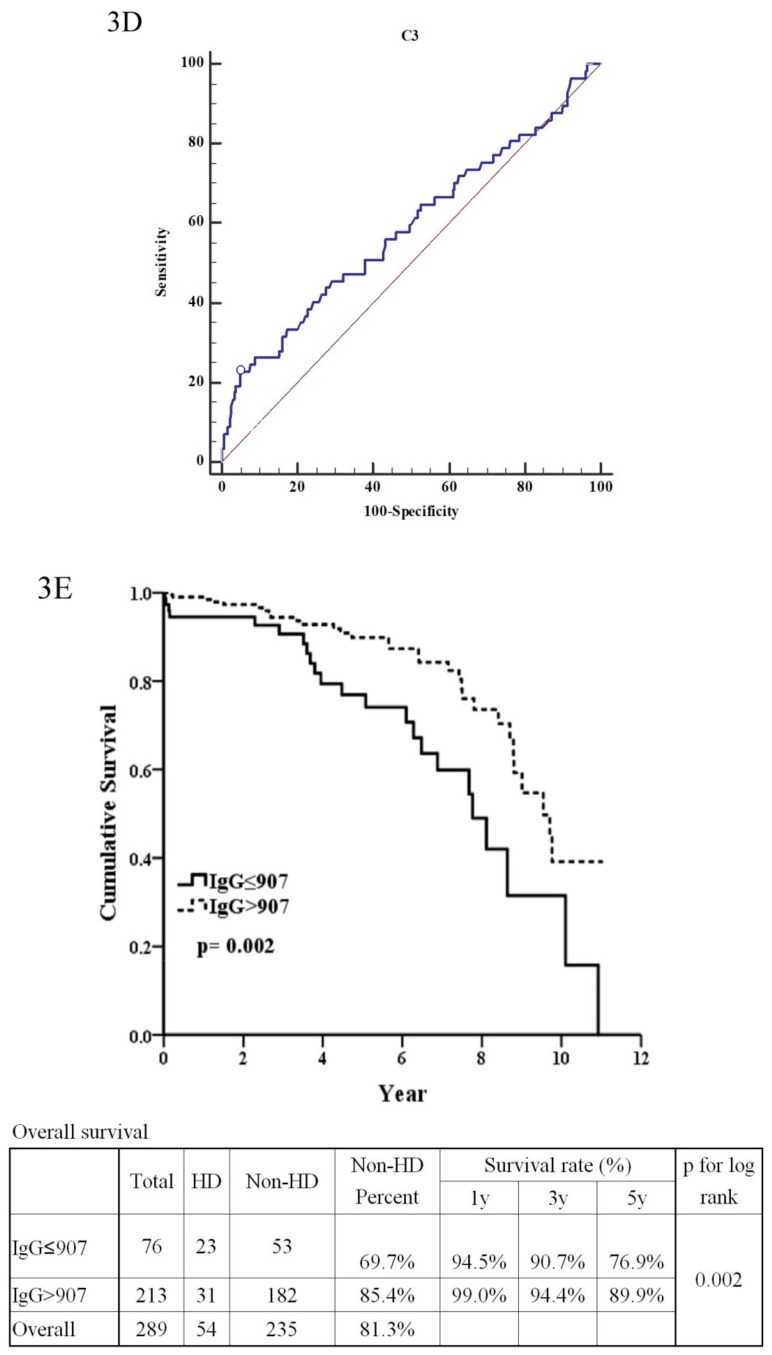

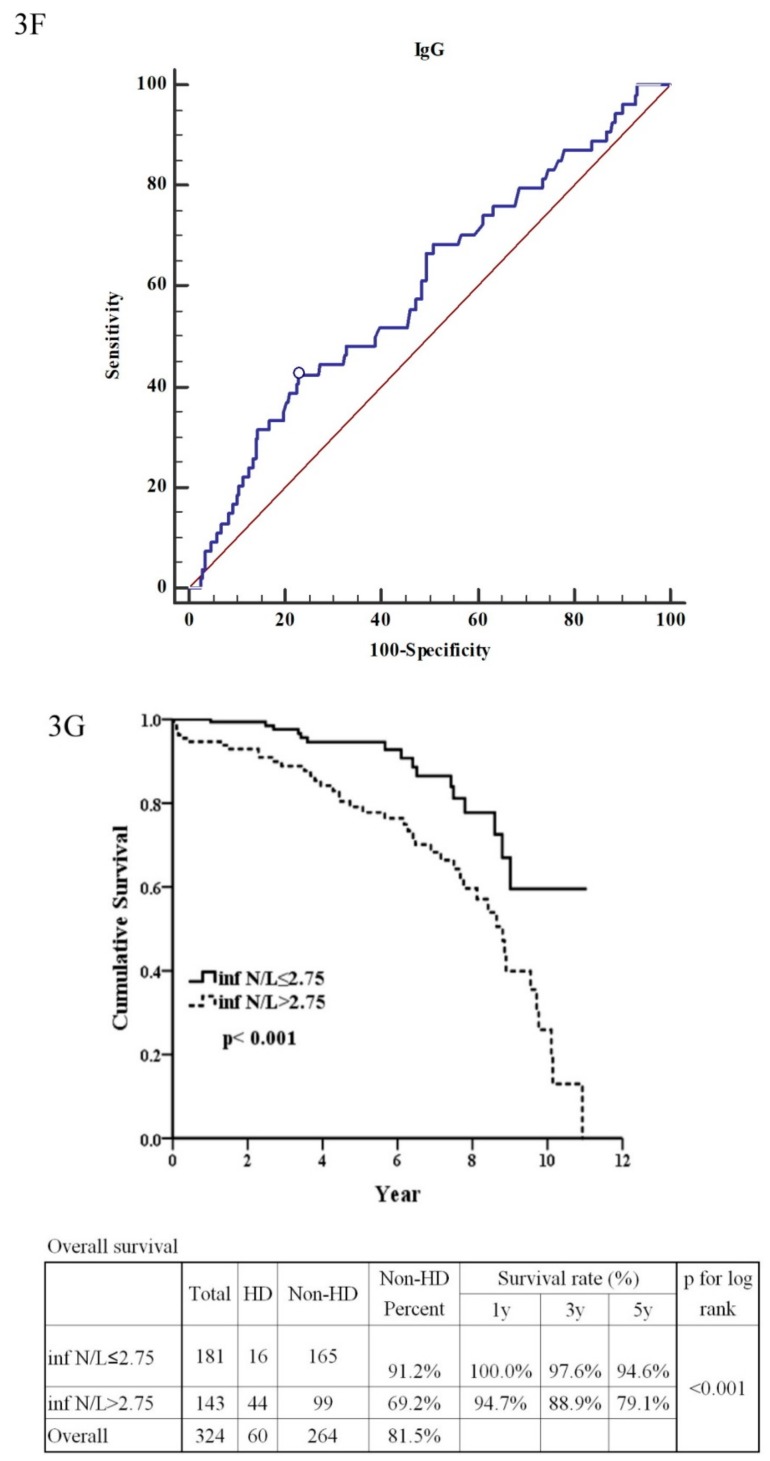

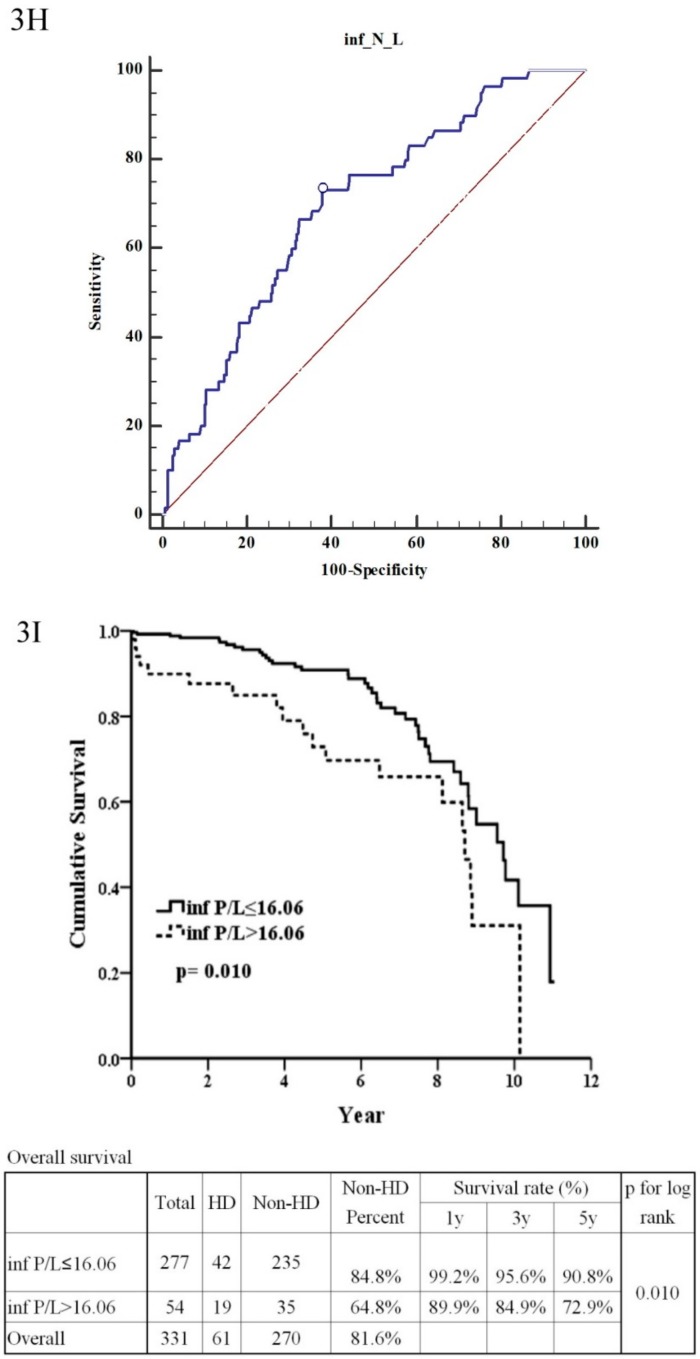

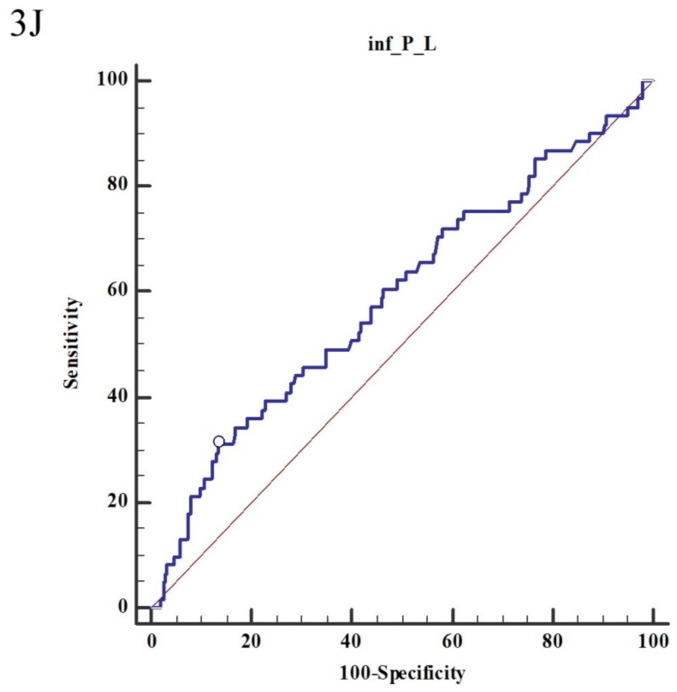
Predicting renal survival of IgA nephropathy with IgA level, C3 level, IgG level, ratio of neutrophil/lymphocyte, and ratio of platelet/lymphocyte, and Kaplan–Meier curve. (**3A**) Renal survival between IgA ≤ 350 or >350 mg/dL without significance. (**3B**) IgA ≤ 350 mg/dL did not predict worse renal survival with poor power (AUC = 0.550 sensitivity = 72.73%, specificity = 41.37%). (**3C**) Renal survival between C3 ≤ 79.7 mg/dL or >79.7 mg/dL differed significantly. (*p* = 0.001). (**3D**) C3 ≤ 79.7 mg/dL predicted worse renal outcome with fair power. (AUC = 0.587, sensitivity = 22.81%, specificity = 94.96%). (**3E**) Renal outcome in IgG ≤ 907 or >907 mg/dL differed significantly (*p* = 0.002). (**3F**) IgG ≤ 907 mg/dL predicted worse renal survival with fair power (AUC = 0.596, sensitivity = 42.59%, specificity = 77.22%). (**3G**) Neutrophil-to-lymphocyte ratio > 2.75 predicted worse renal survival (*p* < 0.001). (**3H**) Neutrophil-to-lymphocyte ratio > 2.75 predicted worse renal outcome with good power. (AUC = 0.696, sensitivity = 73.33%, specificity = 62.17%). (**3I**) Platelet-to-lymphocyte ratio > 16.06 predicted worse renal survival significantly (*p* = 0.010). (**3J**) Platelet-to-lymphocyte ratio > 16.06 predicted worse renal survival with fair power (AUC = 0.590, sensitivity = 31.15%, specificity = 86.45%).

As in Figure 1C, stage 1–3 of Haas classification had better renal outcome than stage 4–5 of Haas classification (*p* < 0.001). As seen in Table 2, the univariate analysis showed that risk factors for poor renal outcome were stage 4–5 of the Haas classification (HR = 3.67, *p* < 0.001), crescent formation (HR = 2.05, *p* = 0.001), poor baseline renal function (HR = 1.02 and *p* < 0.001 for higher BUN; HR = 1.14 and *p* < 0.001 for higher serum creatinine; HR = 0.95, *p* < 0.001 for higher eGFR ), hyperuricemia (HR = 1.30, *p* < 0.001), higher proteinuria (HR = 1.13, *p* < 0.001), IgG ≤ 907 (HR = 2.29, *p* = 0.003), C3 ≤ 79.7 (HR = 2.76, *p* = 0.002), a higher C4 (HR = 1.02, *p* = 0.026), NLR > 2.75 (HR = 2.92, *p* < 0.001), PLR ≥ 16.06 (HR = 2.02, *p* = 0.012), and daily proteinuria (HR = 1.12, *p* < 0.0001). The multivariate analysis showed risk factors for poor renal function were poor baseline renal function (HR = 0.88, *p* = 0.001 for higher eGFR), C3 ≤ 79.7 (HR = 17.27, *p* = 0.003), and a higher C4 (HR = 1.11, *p* = 0.014). There were significant differences for renal survival between C3 ≤ 79.7 or 79.7 (*p* = 0.001, Figure 3C), IgG ≤ 907 or >907 (*p* = 0.002, Figure 3E), NLR > 2.75 ≤ 2.75 (*p* < 0.001, Figure 3G), and PLR ≥ 16.06 or <16.06 (*p* = 0.010, Figure 3I). The statistically significant predictive power for renal outcome was C3 ≤ 79.7 (AUC = 0.587, sensitivity = 22.81%, specificity = 94.96%, *p* = 0.001) (Figure 3D), IgG ≤ 907 (AUC = 0.596, sensitivity = 42.59%, specificity = 77.22%, *p* = 0.002) (Figure 3F), NLR > 2.75 (AUC = 0.696, sensitivity = 73.33%, specificity = 62.17%, *p* < 0.001) (Figure 3H) and PLR ≥ 16.06 (AUC = 0.590, sensitivity = 31.15%, specificity = 86.45%, *p* = 0.010) (Figure 3J). 

## 4. Discussion

Classification of renal pathology has been well outlined through Oxford classification, where 22 validation studies have been published. The Oxford classification, MEST score, is still under revision and update by the Working Group of the International IgA Nephropathy Network [12]. In other words, it remains impossible to predict renal outcome while depending only on this pathological classification [9]. Some limitations to Oxford classifications have been reported [9], including those which are not suitable to more extreme presentations of the disease (proteinuria less than 0.5 g per day or eGFR < 30 mL/min/1.732 m^2^), along with those patients whose follow-up was less than 1 year. As for the older classification, Haas classification, it is on a decline compared to the Oxford classification. One criticized issue for Haas classification is the use of subjective and vague terminology such as “maybe” and “more than”, which reduces their reproducibility [7]. In addition, the Haas classification did not specify mesangial and endocapillary hypercellularity. However, the ability of both the Haas and the Oxford classifications for prediction of renal outcome are comparable (*p* = 0.348 by Harrell’s C statistics) [7]. Therefore, we chose the Haas classification in this cohort. In this study, the background renal status (including proteinuria and GFR) was poorer in the moderate-to-advanced Haas classification. Compared to stage 1–3, stage 4–5 of the Haas classification was better able to significantly predict a worse renal outcome (HR = 3.67), according to univariate analysis. In multivariate analysis, the baseline renal function still exhibited predictive power for renal outcome. The background renal function was strongly associated with the Haas classification (*p* < 0.05). This study is the first to prove that the Haas classification is useful for prediction of renal outcome in an Asian population. Different from the Oxford classification, which has been validated in many studies, our study is the first one to validate the predictive power of the Haas classification within the Asian race. 

Typically, IgAN is induced by the IgA-induced activation of mesangial cells and local complement activation. Polymeric IgA stimulates a phenotypic transformation in mesangial cells, where the mesangial cells then proliferate and secrete extracellular matrix components [13]. In some studies, the code position of IgG may synergistically cause the development of a proinflammatory phenotype in mesangial cells, followed by influencing both glomerular injury and clinical outcome [14]. Glomerular IgG deposits significantly predicted renal outcome, independent of Oxford and clinical variables (HR = 2.97, *p* = 0.04) [14]. In this cohort, lower serum IgG (≤907 mg/dL) was a risk factor for poor renal outcome (HR = 2.29, *p* = 0.003). The lower serum IgG may be due to the deposits in the glomeruli. Also, decline of IgG was also due to its loss in the urine (more significantly was higher proteinuria), and increase in its rate of catabolism [15,16]. The diagnostic power for a poorer renal outcome (IgG ≤ 907 mg/dL) was only acceptable, but still showed statistical significance (AUC = 0.596, sensitivity = 42.59%, specificity = 77.22%). As for the complement activation, C3 was reported to be co-deposited with IgA in over 90% of patients with IgAN [17]. Our study was also consistent with this phenomenon. Lower serum C3 (≤79.7 mg/dL) was risky for experiencing a worse renal outcome (HR-2.76, *p* = 0.002), with fair diagnostic power (AUC = 0.587, sensitivity = 22.81%, specificity = 94.96%). This is consistent with an observational study [18] where hypoC3 significantly predicted renal outcome of double-serum creatinine. In our study, the follow-up duration was much longer. However, a study of propensity score matching did not suggest this result [19]. That may be due to the selection bias and too short follow-up time [19]. Compared to this study, the strength of our study was that we had a much longer follow-up duration. 

The higher the serum C4 level, the worse the long-term renal function was observed in this study, for both univariate (HR = 1.02, *p* = 0.026) and multivariate analysis (HR = 1.11, *p* = 0.014) (even if only a minor risk). The association between C4 and the outcome of IgAN has been much less discussed, but is believed to still be of significance. In the lectin activation pathway, C4 is split into C4a and C4b, which is then converted to C4d [20]. The C4d binds covalently to the endothelial and collagen basement membranes, which thereby leads to the avoidance of antibody activation [20]. The C4d deposition has been reported to be associated with unfavorable histopathological and clinical findings [21]. Additionally, one-fourth of patients demonstrated evidence of lectin pathway activation (glomerular deposition of Mannose-binding lectin (MBL)), whereby these patients exhibited more renal injuries [17]. Mannose-binding lectin is able to bind to polymeric IgA, which causes the activation of both C3 and C4 [22]. In summary, the serum C4 level is also responsible for the pathogenesis of IgAN, even though its role may be minor (increased by 2% or 11% of HR) (univariate or multivariate analysis, respectively). 

An NLR (>2.75) predicts a worse renal outcome (HR = 2.92, *p* < 0.001), while the diagnostic power was good (AUC = 0.696, sensitivity = 73.33%, specificity = 62.17%). This is the first study to indicate that NLR has the ability to predict renal outcome. NLR is a simple and inexpensive laboratory marker for determining inflammatory status, including cardiovascular disease [23], malignancy [24,25,26] and cirrhosis [26]. The normal values for patients in good health are between 0.78 and 3.53 [27]. Baseline NLR may have a predictive value for renal prognosis in both granulomatosis with polyangiitis [28] and lupus nephritis [29]. NLR is also a potential indicator for prognosticating systemic involvement in adult IgA vasculitis (formerly Henoch–Schonlein purpura) [30,31] with the strongest diagnostic value [30]. Kar et al. cited that disordered neutrophil activation could be relevant to the pathogenesis of IgAN due to the increased expression of complement 3 receptors on neutrophils from patients with IgAN [32]. An increased oxidative metabolism of neutrophils in patients with IgAN was also suspected [32]. There is accumulating evidence that neutrophils are involved in inflammatory injury in IgAN [33]. Also, an increased NLR may be due to the lower lymphocyte count. It has been proposed that while under physiologic stress, there is a release of cortisol, where this increase in cortisol causes lymphopenia [34]. Similarly, PLR is also considered an inflammatory marker used as a prognostic factor in thrombotic events, inflammatory diseases, and malignancies [35,36]. Recently, it has been associated with all-cause of mortality in geriatric patients diagnosed with chronic kidney disease [37], associated with higher C-reactive protein in patients with end-stage renal disease [38], and erythropoietin resistance [39]. In this study, patients with a PLR > 16.06 were labeled as having risky-to-poor renal outcomes (HR = 2.02, *p* = 0.012), even if there were poor diagnostic capabilities (AUC = 0.590, sensitivity = 31.15%, specificity = 86.45%), but with statistical significance. This is the first study to highlight that PLR can predict renal outcome in IgAN. Platelets can interact with various types of immune cells, including endothelial cells, dendritic cells, T-cells, neutrophils, and mononuclear phagocytes. These interactions may initiate and exacerbate the inflammation in the arterial wall [40]. Therefore, we suggest routine checks which can be identified easily, NLR and PLR, in order to predict renal outcome. More detailed research is still required for better understanding the associated mechanism of NLR and PLR in IgAN.

There were several limitations in this study. Firstly, the subjects were chosen from only a single center. However, in this center, we have performed more than 8000 renal biopsies within the past 30 years. Secondly, no genetic associations were analyzed. However, the genetic predictive data are often conflicting and confounded by the population which is being studied (population stratification) [41,42,43]. Thirdly, there were data for treatment. However, the standard treatment for IgAN is consistent in this medical center. Angiotensin-converting enzyme inhibitors (ACEis) or angiotensin II receptor blockers (ARBs) were prescribed for all patients with IgAN. This immunosuppressive therapy will be given for those experiencing a progressively declining GFR, persistent proteinuria above 1 g/day after maximal ACEis or ARBs. Fourthly, we did not measure aberrantly galactosylated IgA1 levels, which had been found to be correlated with IgAN prognosis [44]. However, in clinical practice, we did not measure this marker. Therefore, our study is a study in the real world. Finally, some additional potentially modifiable risk factors, such as hypertension [45,46,47], hypertriglyceridemia and hyperuricemia [48], and smoking [49] were not found as risk factors in this study.

## 5. Conclusions

In summary, our study has demonstrated that the Haas classification is a useful system with predictive value in Asian populations. Lower serum IgG (≤907 mg/dL) and serum C3 (≤79.7 mg/dL) levels were found to be risk factors for poor renal outcome. Additionally, this is the first study to point out that serum C4 levels, an NLR > 2.75, and a PLR > 16.06 may indicate a poor renal outcome.

## Figures and Tables

**Table 1:** Demographic characteristics of IgA nephropathy by Haas’ classification.

	Total (*n* = 272)		Stage 1–2 (*n* = 66)		Stage 3–5 (*n* = 206)		*p*-Value
	*n*	±SD or %	*n*	±SD or %	*n*	±SD or %	
Male	151	55.5%	34	51.5%	117	56.8%	0.543
Age (years old)	39.25	±15.39	40.21	±15.50	38.95	±15.38	0.498
Crescent	44	16.2%	3	4.5%	41	19.9%	0.006 **
SBP (mmHg)	131.53	±18.49	131.74	±21.27	131.47	±17.57	0.833
DBP (mmHg)	83.69	±13.95	82.48	±14.50	84.07	±13.78	0.411
Body height (cm)	164.35	±9.11	163.38	±9.68	164.69	±8.92	0.497
Body weight (kg)	65.43	±12.71	65.90	±13.61	65.28	±12.44	0.493
BUN (mg/dL)	26.90	±23.43	19.49	±12.85	29.23	±25.47	<0.001 **
Creatinine (mg/dL)	2.01	±2.31	1.23	±0.67	2.26	±2.58	0.001 **
eGFR (min/min/1.732 m^2^)	64.65	±40.07	73.96	±32.79	61.69	±41.76	0.006 **
Blood WBC (/cumm)	7853.44	±2368.22	7930.89	±2638.71	7828.63	±2281.19	0.612
Blood RBC (/cumm)	4.19	±0.84	4.37	±0.72	4.13	±0.86	0.063
Hemoglobin (g/dL)	12.49	±2.17	13.12	±1.81	12.28	±2.24	0.008 **
Neutrophil (%)	63.78	±10.03	63.31	±9.34	63.92	±10.25	0.607
Lymphocyte (%)	26.40	±8.99	27.27	±9.16	26.12	±8.94	0.426
Platelet (10^3^/cumm)	253.80	±93.91	253.91	±60.35	253.76	±102.48	0.562
Uric acid (mg/dL)	7.35	±2.12	6.81	±2.01	7.51	±2.13	0.016 *
Na (meq/L)	140.08	±3.14	140.33	±3.23	140.00	±3.11	0.408
K (meq/L)	4.25	±0.53	4.15	±0.41	4.28	±0.56	0.145
Ca (mg/dL)	8.54	±1.02	8.83	±0.81	8.45	±1.07	0.001 **
P (mg/dL)	3.90	±1.05	3.58	±0.59	3.99	±1.14	0.037
Mg (mg/dL)	2.34	±0.44	2.16	±0.45	2.39	±0.43	0.264
Albumin (g/dL)	3.75	±0.60	3.96	±0.62	3.68	±0.59	<0.001 **
Total protein (gm/dL)	6.62	±0.85	6.84	±0.77	6.55	±0.86	0.001 **
GOT (U/L)	24.34	±20.42	27.57	±21.79	23.34	±19.94	0.124
GPT (U/L)	23.42	±19.99	26.44	±25.30	22.47	±17.98	0.385
Total cholesterol (mg/dL)	196.28	±51.81	195.98	±68.29	196.38	±45.44	0.397
Triglyceride (mg/dL)	137.67	±90.43	129.84	±79.09	140.09	±93.73	0.272
LDL (mg/dL)	118.67	±40.84	106.41	±30.79	122.69	±42.95	0.005 **
HDL (mg/dL)	56.39	±19.80	57.67	±23.21	55.95	±18.56	0.668
Fasting glucose (mg/dL)	92.72	±17.36	96.06	±25.13	91.73	±14.20	0.147
Postprandial glucose (mg/dL)	129.11	±68.81	102.00	±4.24	132.50	±72.49	1.000
HbA1c (%)	5.58	±0.87	5.72	±1.02	5.54	±0.82	0.289
IgG (mg/dL)	1103.04	±337.82	1185.85	±341.74	1074.34	±332.63	0.897
IgA (mg/dL)	361.21	±136.63	361.40	±135.43	361.14	±137.40	0.556
IgM (mg/dL)	114.52	±53.12	118.97	±56.25	113.01	±52.10	0.930
IgE (mg/dL)	208.68	±358.03	203.06	±428.18	210.49	±336.55	0.068
C3 (mg/dL)	110.75	±24.27	115.54	±23.51	109.15	±24.36	0.061
C4 (mg/dL)	29.82	±10.77	27.92	±10.46	30.46	±10.82	0.398
Negative for HBV	215	79.0%	52	78.8%	163	79.1%	0.428
Negative for HCV	244	89.7%	61	92.4%	183	88.8%	0.249
ANA (≥1:160)	22	8.4%	6	9.4%	16	8.0%	0.921
dsDNA	19.26	±17.07	24.64	±25.93	17.46	±12.58	0.645
ANCA	5	3.2%	1	2.6%	4	3.4%	1.000
MPO	11.60	±31.83	35.40	±65.75	6.31	±18.04	0.414
PR3	3.67	±3.51	2.73	±2.53	3.93	±3.76	0.614
Urinary albumin (mg/dL)	64.18	±86.65	17.24	±27.79	74.13	±91.77	0.022 *
24 h proteinuria (g/day)	2.11	±2.15	1.25	±1.84	2.38	±2.17	<0.001 **
Urine creatinine (mg/dL)	100.48	±67.95	115.89	±71.08	95.45	±66.31	<0.001 **
Urine PCR (mg/mg)	1.91	±2.57	0.97	±1.17	2.21	±2.82	0.021 *
Urine ACR (mg/g)	854.05	±1145.90	184.75	±248.90	818.85	±955.30	0.009 **

Chi-square test. Mann–Whitney U test. * *p* < 0.05, ** *p* < 0.01.

**Table 2:** Cox regression models for renal survival via univariate and multivariate analysis.

	Univariate			Multivariate		
	Hazard Ratio	95%CI	*p*-Value	Hazard Ratio	95%CI	*p*-Value
Sex (male/female)	1.14	(0.68–1.91)	0.631			
Age, year	1.03	(1.01–1.05)	0.001 **			
Crescent (with/without)	2.05	(1.12–3.75)	0.019 *			
BUN	1.02	(1.01–1.02)	<0.001 **			
Serum creatinine	1.14	(1.09–1.20)	<0.001 **			
eGFR	0.95	(0.94–0.97)	<0.001 **	0.88	(0.82–0.95)	0.001 **
Uric acid	1.30	(1.17–1.44)	<0.001 **	0.93	(0.64–1.36)	0.716
Albumin	0.44	(0.31–0.62)	<0.001 **	0.80	(0.22–2.85)	0.728
Total protein	0.52	(0.40–0.68)	<0.001 **	0.95	(0.44–2.08)	0.903
LDL	1.00	(0.99–1.01)	0.735			
Urine PCR	1.13	(1.08–1.19)	<0.001 **			
IgG (≤907:>907)	2.29	(1.33–3.96)	0.003 **	0.90	(0.20–4.14)	0.897
IgA (>350:≤350)	1.07	(0.62–1.85)	0.806			
C3 (>79.7:≤79.7)	2.76	(1.46–5.23)	0.002 **	17.27	(2.62–113.81)	0.003 **
C4	1.02	(1.00–1.04)	0.026 *	1.11	(1.02–1.21)	0.014 *
Inflammatory marker						
Neutrophil/Lymphocyte ratio (>2.75:≤2.75)	2.92	(1.65–5.19)	<0.001 **	0.75	(0.18–3.09)	0.690
Platelet/Lymphocyte ratio (≤16.06:>16.06)	2.02	(1.17–3.49)	0.012 *	1.41	(0.33–5.91)	0.641
24 h proteinuria	1.12	(1.05–1.19)	<0.001 **			
Haas’ classification (stage 1–3:stage 4–5)	3.67	(1.89–7.13)	<0.001 **			

Cox proportional hazard regression. * *p* < 0.05, ** *p* < 0.01.

## Abbreviations

IgAN	Immunoglobulin A nephropathy
ESRD	End-stage renal disease
SBP	Systolic blood pressure
DBP	Diastolic blood pressure
BUN	Blood urea nitrogen
eGFR	Estimated glomerular filtration rate
WBC	White blood cell
RBC	Red blood cell
GOT	Glutamate oxaloacetate transaminase
GPT	Glutamate-pyruvate transaminase
LDL	Low-density lipoprotein
HDL	High-density lipoprotein
NLR	Neutrophil-to-lymphocyte ratio
PLR	Platelet-to-lymphocyte ratio
ANA	Anti-nuclear Ab
anti-dsDNA	Anti-double stranded DNA
ANCAs	Anti-neutrophil cytoplasmic antibodies
MPO	Proteinase 3 (PR3) and myeloperoxidase
ACEis	Angiotensin-converting enzyme inhibitors
ARBs	Angiotensin II receptor blockers
